# Fluoxetine Protection in Decompression Sickness in Mice is Enhanced by Blocking TREK-1 Potassium Channel with the “spadin” Antidepressant

**DOI:** 10.3389/fphys.2016.00042

**Published:** 2016-02-16

**Authors:** Nicolas Vallée, Kate Lambrechts, Sébastien De Maistre, Perrine Royal, Jean Mazella, Marc Borsotto, Catherine Heurteaux, Jacques Abraini, Jean-Jacques Risso, Jean-Eric Blatteau

**Affiliations:** ^1^Institut de Recherche Biomédicale des Armées, Equipe Résidante de Recherche Subaquatique OpérationnelleToulon, France; ^2^UFR STAPS, Laboratoire Motricité Humaine Education Sport Santé, Université du Sud Toulon VarLa Garde, France; ^3^Hôpital d'Instruction des Armées, Service de Médecine Hyperbare et Expertise PlongéeToulon, France; ^4^Centre National de la Recherche Scientifique and Université de Nice Sophia Antipolis, Institut de Pharmacologie Moléculaire et Cellulaire, UMR 7275Valbonne, France; ^5^Département d'Anesthésiologie, Université LavalQuébec, QC, Canada; ^6^Faculté de Médecine, Université de Caen NormandieCaen, France

**Keywords:** bubble, decompression sickness, diapedesis, kcnk2, TREK-1, diving, depression, capillary leak

## Abstract

In mice, disseminated coagulation, inflammation, and ischemia induce neurological damage that can lead to death. These symptoms result from circulating bubbles generated by a pathogenic decompression. Acute fluoxetine treatment or the presence of the TREK-1 potassium channel increases the survival rate when mice are subjected to an experimental dive/decompression protocol. This is a paradox because fluoxetine is a blocker of TREK-1 channels. First, we studied the effects of an acute dose of fluoxetine (50 mg/kg) in wild-type (WT) and TREK-1 deficient mice (knockout homozygous KO and heterozygous HET). Then, we combined the same fluoxetine treatment with a 5-day treatment protocol with spadin, in order to specifically block TREK-1 activity (KO-like mice). KO and KO-like mice were regarded as antidepressed models. In total, 167 mice (45 WT_cont_ 46 WT_flux_ 30 HET_flux_ and 46 KO_flux_) constituting the flux-pool and 113 supplementary mice (27 KO-like 24 WT_flux2_ 24 KO-like_flux_ 21 WT_cont2_ 17 WT_no dive_) constituting the spad-pool were included in this study. Only 7% of KO-TREK-1 treated with fluoxetine (KO_flux_) and 4% of mice treated with both spadin and fluoxetine (KO-like_flux_) died from decompression sickness (DCS) symptoms. These values are much lower than those of WT control (62%) or KO-like mice (41%). After the decompression protocol, mice showed significant consumption of their circulating platelets and leukocytes. Spadin antidepressed mice were more likely to exhibit DCS. Nevertheless, mice which had both blocked TREK-1 channels and fluoxetine treatment were better protected against DCS. We conclude that the protective effect of such an acute dose of fluoxetine is enhanced when TREK-1 is inhibited. We confirmed that antidepressed models may have worse DCS outcomes, but concomitant fluoxetine treatment not only decreased DCS severity but increased the survival rate.

## Introduction

In this study, fluoxetine and the new antidepressant spadin were used to find a treatment strategy against decompression sickness.

Circulating bubbles cause cell damage (Vallee et al., 2013), prothrombotic phenomena, ischemia, and diapedesis (Dutka et al., 1989; Zamboni et al., 1989, 1992, 1993; Dal Palu and Zamboni, 1990; Helps and Gorman, 1991). This inflammation can spread systemically and may degenerate into a vicious cycle, ending in multiple organ failure (Jacey et al., 1976; DeGirolami and Zivin, 1982; Ersson et al., 1998). Spinal cord and brain neurological damage underlie the most serious symptoms of decompression sickness (DCS; Gempp et al., 2008). These symptoms result from circulating bubbles generated by a pathogenic decompression, following a dive for example. Even after standard treatment with hyperbaric oxygen, 20–30% of victims suffer from sequelae after a neurological DCS (Blatteau et al., 2011). We aimed to establish a potential treatment strategy using an animal model of DCS.

Fluoxetine, the active compound in the antidepressant Prozac™, prevents the reuptake of serotonin (5-hydroxytryptamine, 5-HT) by inhibiting serotonin transporters (SERT) located in neurons, platelets (Lesch et al., 1993), and leukocytes (Faraj et al., 1994; Lima and Urbina, 2002; Yang et al., 2007). SERT increases the concentration of circulating serotonin (Brenner et al., 2007). When used in a single high dose, fluoxetine is also believed to mediate neuroprotection (Pariente et al., 2001; Chollet et al., 2011; Taguchi et al., 2012) by inhibiting NMDA-R (Vizi et al., 2013), regulating inflammatory effects (Kubera et al., 2001; Jin et al., 2009; Lim et al., 2009) and algesia (Kostadinov et al., 2014). We have previously demonstrated that WT mice treated with fluoxetine are more resistant to DCS and that fluoxetine inhibits the inflammatory process by reducing the level of circulating IL-6, a pro-inflammatory cytokine (Blatteau et al., 2012).

TREK-1, the product of the *kcnk2* gene, regulates cell excitability and prevents neuron death by inhibiting NMDA-dependent glutamatergic excitotoxicity induced by ischemia (Franks and Honore, 2004; Heurteaux et al., 2004; Buckler and Honore, 2005; Honore, 2007; Dedman et al., 2009). The mechanosensitive TREK-1 channel (Franks and Honore, 2004) is activated by a mechanical deformation of the cellular membrane, for example by a deformation induced by air depression (Heurteaux et al., 2004). Additionally, in mice, decompression-induced desaturation also activates TREK-1. Consequently, KO (“KO” is used in the mean of “TREK-1^−/−^ mice”) mice are more sensitive to DCS than WT (TREK-1^+/+^) mice (Vallee et al., 2012). TREK channel activity is also under the control of several different mechanisms acting either on channel trafficking and surface density or directly on gating properties (Noel et al., 2011). Furthermore, KO mice display a depression-resistant phenotype, similar to chronic fluoxetine-treated mice (Heurteaux et al., 2006).

We have shown that the opening of TREK-1 channels is protective in DCS, suggesting that TREK-1 channel activity limits ischemia-induced glutamatergic toxicity (Vallee et al., 2012). The TREK-1 channel is directly inhibited by fluoxetine (Heurteaux et al., 2006; Bogdan et al., 2011; Sandoz et al., 2011) and by spadin, a new antidepressant that internalizes the channel after a 5-days treatment and consequently abolish channel activity (Mazella et al., 2010; Moha Ou Maati et al., 2011), so the question arises as to whether acute fluoxetine treatment could be significantly more efficient when the TREK-1 channel is impaired: we suggest if less fluoxetine can link to TREK-1, the fluoxetine anti-inflammatory effect should be enhanced in a dose-dependent manner, although the neuroprotection afford by the TREK-1 activity could be loss. Conversely, we wondered whether mice pre-treated with an anti-depressant drug, that impairs TREK-1 activity, would be sensitized to DCS.

In this study, we wanted to obtain a better understanding of the protection afforded by fluoxetine in our DCS model. We aimed to block the fluoxetine binding on TREK-1, and therefore to promote other fluoxetine pathways, resulting in a better efficiency for the inhibition of the NMDA-R or the regulation of interleukin releases for example (**Figure 2**).

Before the exposure to the pathogenic decompression, we gave an acute dose of fluoxetine on wild-type (WT: TREK-1^+/+^) mice and on mice to whom TREK-1 disappeared from the membrane surface. We therefore used TREK-1 knockout (KO: TREK-1^−/−^) and heterozygous (HET: TREK-1^+/−^) mice, or 5 days of treatment with spadin resulting in KO-like (TREK-1^−/−*like*^) mice, to inhibit TREK-1 channel activity. KO and KO-like mice were considered the antidepressed models. We obviously did not use chronic fluoxetine (more than 21 days are necessary) on WT mice to obtain an antidepressive state, followed by an acute dose of fluoxetine; while of interest, this would not allow us to reach a conclusion.

## Materials and methods

### Animals and ethical statement

All procedures involving experimental animals were in line with European Union rules (Directive 2010/63/EU) and French law (Decree 2013/118). The ethics committee of the Institut de Recherche Biomédicale des Armées approved this study. According to our animal care committee, a scoring system inspired by the Swiss veterinary guidelines was implemented to ensure the welfare of animals. A dedicated observer scored (from 0 to 3) the stress or pain relating to some criteria for each animal, and then completed a sheet (Supplementary Data). Pain of degree 3 (very painful) in one case or a total score of 12 in the table were the ethical endpoints. On this sheet, the most commonly found were: vocalizing, aggression or withdrawn behavior, reduction in exploratory behavior, licking, closed eyes, tears, bubbles in the eyes, high respiratory rate, runny nose, fur bristling, labored breathing, convulsions, paralysis, difficulty moving, and problems with the fore or rear limbs (classified as motor disorders). In this study, no score reached 12 and there was no need to cull the animal based on these criteria. Actually, mice displaying degree 3 convulsions died very rapidly. At the end of the experiment, mice were anesthetized first with halothane (5% in oxygen, Halothane, Belamont, France) in order to gain time and to minimize stress, and then with an intraperitoneal injection of a mixture of 16 mg/kg xylazine (Rompum® 2%, Bayer Pharma) and 100 mg/kg ketamine (Imalgène® 1000, Laboratoire Rhône). Our investigator (NV) is associated with agreement number 83.6 delivered by the Health and Safety Directorate of our department, as stated in the French rules R.214-93, R-214-99, and R.214-102. Mice were housed in an accredited animal care facility. Mice kept were in group cages both during rest and during the experiments and maintained on a regular day (6:00 a.m.–6:00 p.m.)/night (12 h) cycle. Food (*AO3, UAR*) and water were provided *ad libitum* and the temperature was kept at 22 ± 1°C.

In TREK-1^−/−^ mice, the gene encoding the TREK-1 channel was knocked out by Cre-Lox recombination (Heurteaux et al., 2004). The comparator animals were analogous C57Bl/6 mice (Charles River Laboratory, Arbresle, France). In order to preclude phenotypic variation between the different strains (Sato et al., 2006), crosses were made every 11 generations. Wild-type (WT, TREK-1^+/+^) heterozygous (HET, TREK-1^+/−^) and knockout (KO, TREK-1^−/−^) mice were produced for this study. To avoid fluctuations due to female hormonal cycles, only males were used in this study.

### Flux-pool

**Figure 1:**
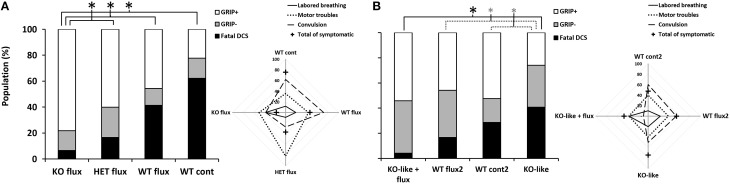
**Symptoms in different groups of mice 30 min after pathogenic decompression as a function of genotype or drug treatment or both. (A)** The flux-pool. **(B)** The spad-pool. Histogram: black blocks represent the percent of mice that died following the dive (Fatal DCS); gray represents mice that failed at least one grip test (sensory motor test for forelimbs) (Grip−); white represents the proportion of mice that passed both grip tests (Grip+). Radar Chart: percentage of mice displaying a type of symptom in a population; cross represents the total (in percent) of Grip− mice, considering that a mouse can present several symptoms at the same time. WT, wild-type; KO, knockout; HET, heterozygous; KO-like, mice treated for 5 days with spadin. Flux, fluoxetine; cont and cont2, control (no active molecules). Black ^*^ denotes *p* < 0.05 between groups. Gray ^*^ denotes *p* < 0.10 (trend) between groups.

In total, 167 mice (6–9 week-old) constituting the flux-pool were exposed to compressed air to induce DCS. The mice were randomly divided into four groups and numbered (Figure 1): 46 for the wild-type group treated with fluoxetine (WT_flux_), 30 for the heterozygous group treated with fluoxetine (HET_flux_), 46 for the knockout group treated with fluoxetine (KO_flux_), and 45 wild-type controls (WT_cont_).

Fluoxetine (50 mg/kg) was administered by gavage to experimental animals as an oral solution (Prozac™ 20 mg/5 ml, oral solution bottle of fluoxetine hydrochloride, Lilly Laboratories, France) 18 h before hyperbaric exposure, while the wild type control group (WT_cont_) received a similar saccharine fluoxetine-free solution (7.4 g/kg). This high dose of fluoxetine was determined on the basis of previous results from a mouse model of ischemia (Jin et al., 2009; Blatteau et al., 2012; Taguchi et al., 2012).

### Spad-pool

In total, 113 mice (strain C57Black/6, 6–9 weeks of age, Harlan laboratories, Gannat, France) constituted the spad-pool and were used to replicate the experiment performed with the flux-pool, but substituting TREK-1 KO mice with spadin-induced KO mice (KO-like mice). To generate KO-like mice, mice were treated for 5 days at 9:00 a.m. with intraperitoneal injections of spadin (100 μg/Kg) in a bolus of 100 μL of NaCl 0.9%. The mixture for the acute fluoxetine treatment (50 mg/kg; 1.5 mg per mouse) was obtained from 20 mg capsules (fluoxetine hydrochloride Prozac™, Lilly Laboratories, France) diluted in 300 μL of a solution of NaCl 9% and gum arabic 5%. It was injected intraperitoneally 18 h before the end of the hyperbaric exposure (p.m. 5:00).

The first group (WT_cont2_, *n* = 21), for control, received the equivalent 5 days of treatment (100 μL per day of NaCl 0.9%, i.p.) plus the vehicle (300 μL of NaCl 0.9% with gum arabic 5%) corresponding to the acute dose of fluoxetine. A second group (KO-like = WT_spadin 5d_, *n* = 27) received a daily dose of spadin for 5 days plus the vehicle of the acute fluoxetine treatment. A third group (WT_flux2_, *n* = 24) received an acute i.p. injection of fluoxetine plus the analogous 5 days-treatment without spadin. A fourth group (KO-like_flux_ = WT_flux+spadin 5d_, *n* = 24) received 5 days of treatment with spadin and the acute dose of fluoxetine. A fifth group (WT_no dive_, *n* = 17) not submitted to the hyperbaric protocol received the same injections as WT_cont2_ mice. Batches were mixed for the hyperbaric protocol.

### Hyperbaric procedure

Hyperbaric Exposure Began at 12:30 p.m.

Each mouse was weighed 30 min before the dive. Samples of 20 mice (10 per cage) from the flux-pool or batches of 6–8 mice from the spad-pool were subjected to the hyperbaric protocol in a 200-l tank fitted with three observation ports. The mice were free to move around the cage.

The compression protocol involved two ramps of pressure increase, first at 0.1 atm/min up to 1 atm, followed by 1 atm/min up to 9 atm; 9 atm corresponds to the pressure where animals were kept for 45 min before decompression. The decompression rate was 60 atm/min up to the surface. Compression and decompression were automatically controlled by a computer linked to an analog/digital converter (NIUSB-6211, National Instrument, USA) with two solenoid valves (Belino LR24A-SR, Switzerland) and a pressure transmitter (Pressure Transmitter 8314, Burket Fluid Control System, Germany). The software was programmed on a DasyLab (DasyLab National Instrument, USA) by our engineer. The software also controlled the temperature and oxygen rate. Compressed air was generated using a diving compressor (Mini Verticus III, Bauer Comp, Germany) coupled to a 100-l tank at 300 bars. The oxygen analyzer was based on a MicroFuel electrochemical cell (G18007 Teledyne Electronic Technologies/Analytical Instruments, USA). The temperature inside the tank was monitored using a platinum-resistance temperature probe (Pt 100, Eurotherm, France).

Water vapor and CO_2_ produced by the animals were captured with soda lime (<300 ppm captured by the soda lime) and seccagel (relative humidity: 40–60%). Gases were mixed by an electric fan. The day-night cycle was respected throughout.

### Behavioral and clinical observations

At the end of the decompression time, mice were observed for 30 min. All signs were recorded together with their time of onset: labored breathing, convulsions or death. Paralysis, difficulty moving and problems with the fore or rear limbs were classified as motor deficits.

The grip test, a motor/sensory test adapted from Hall et al. (Hall, 1985), was used to quantify forelimb involvement 15 and 30 min after the end of decompression. The mouse was placed in the middle of a 60 cm-long cord suspended at a height of 40 cm hanging from its fore paws and each performance was timed with a stop-watch, over a test duration of 30 s. Mice which escaped by climbing up and then walking along the cord corresponded to the highest score of 30 s. Mice which failed at least one test were considered as symptomatic (Grip−). The results of this behavioral test were used to define DCS and distinguished the following groups: dead (fatal DCS), mice that failed at least one grip test (Grip−) and mice that passed the grip test (Grip+).

### Anesthesia and sacrifice

All mice were anaesthetized 30 min after surfacing (after grip tests) first with halothane (5% in oxygen, Halothane, Belamont, France) in order to gain time and to minimize stress, and then by intraperitoneal injection of a mixture of 16 mg/kg xylazine (Rompum® 2%, Bayer Pharma) and 100 mg/kg ketamine (Imalgène® 1000, Laboratoire Rhône). Mice in the spad-pool were kept for heart blood sampling for biochemistry and then sacrificed by injecting pentobarbital (200 mg/kg ip, Sanofi Santé, France).

### Blood tests

Blood tests were carried out using an automatic analyzer (ABCvet, SCIL Animal care company, France) on samples taken before the dive and again 30 min afterwards. The second test values were corrected according to the hematocrit variation. Leukocytes, erythrocytes, hematocrit, platelets and Mean Platelet Volume (MPV) were analyzed in 20 μl samples taken from the tip of the tail and diluted in the same volume of 2 mM EDTA (Sigma, France).

Blood biochemistry [Na^+^, K^+^, Ca^2+^, creatinine kinase, glucose, blood urea nitrogen (BUN), creatinine, transaminase, bilirubin, albumin, globulin, total proteins] was conducted with automatic analyzers (Vetscan VS2, Abaxis Veterinary Company, France; Reflovet Plus SCIL Animal Care Company, France; and Accutrend Plus, Roche Diagnostic, USA) on lithium heparin (Sigma, France) blood samples from the heart. Hemolytic samples were rejected.

### Genotyping

For first part of the study (flux-pool), DNA for PCR was extracted from cells from the tip of the tail (5 mm) after overnight digestion at 56°C with protease K (200 μg/ml) (Promega, Charbonnière, France) freshly added in a buffer solution containing 100 mM Tris (pH 8.5), 200 mM NaCl, 5 mM EDTA, and 0.2% SDS. The protease K was then heat-inactivated (95°C for 5–10 min). The lysate was diluted 20-fold in ultrapure water before amplification.

PCR was carried out on 5 μl of lysate added to 20 μl of the reaction mixture. For negative controls, water was substituted for the lysate. The reaction mixture contained a pair of primers (10 pM/μl) [1–2] or [1–3] with an amplification mixture (GoTaq®Green Master Mix 2X, Promega France). DNA primers (MWG-Opéron Biotech, France) corresponding to the loci of interest in the *kcnk2* gene (Primers #1 [5′ GGT GCC AGG TAT GAA TAG AG 3′]; Primers #2 [5′ TTC TGA GCA GCA GAC TTG G 3′]; Primers #3 [5′ GTG TGA CTG GGA ATA AGA GG 3′]) were used with the following thermocycler (MultiGene Gradient, Labnet International, USA) settings: initialization step 94°C/3 min>> [denaturation step 94°C/25 s >> annealing step 61°C/25 s >> elongation step 72°C/35 s] for 35 cycles.

Amplified DNA sequences were resolved by electrophoresis (Biorad Generator, Powerpac 200; 90 V 45 min) on buffered 1.2% Tris acetate EDTA agar gels supplemented with BET for UV detection (Geneflash, Syngene Bioimaging). PCR-detected bands at both 680 bp [1–2] and 1870 bp [1–3] characterize the homozygous wild-type (WT, TREK-1^+/+^), and a single band at 650 bp [1–3] characterizes the homozygous knock-out (KO, TREK-1^−/−^). A pair of bands characterizes heterozygous mice (HET, TREK-1^−/+^).

### Statistical analyses

Individual blood cell count data were calculated as the percentage change from baseline (the measurement before hyperbaric exposure). Numerical data points are expressed as mean and standard deviation. A contingency table was used for independence and association tests coupled with the χ^2^ significance test. Different groups were compared using the Mann-Whitney (MW) test and matched comparisons within groups were analyzed using the Wilcoxon (W) test. Multiple comparisons were performed using the Kruskal-Wallis test followed by the Bonferroni-Dunn *post-hoc* test. The significance threshold was 95% with an α-risk of 5%.

## Results

Flux-pool: 167 mice (91 WT, 30 HET, and 46 KO) were subjected to the hyperbaric protocol to induce DCS. In these groups, mouse weights were similar [weight_WTcont_: mean = 24.0 ± 2.3 g (range: 19.4–29.3 g); weight_WTflux_: mean = 24.2 ± 0.6 g (range: 23.7–25.1 g); weight_HETflux_: mean = 24.5 ± 2.4 g (range: 20.4–29.4 g); weight_KOflux_: mean = 24.4 ± 2.1 g (range: 20.8–30.6 g)] (KW_WTcont/WTflux/HETflux/KOflux_: *n* = 45/46/30/46, α = 0.05, *p* = 0.657).

Spad-pool: 113 mice were used in this pool. Spadin was used to generate TREK-1 KO-like mice. Again, mouse weights were similar [weight _KOlike_: mean = 27.4 ± 2.2 g (range: 23.0–31.5 g); weight _WTflux_: mean = 28.2 ± 2.7 g (range: 23.7–34.4 g); weight _KO-like flux_: mean = 28.7 ± 2.0 g (range: 25.1–33.0 g) weight _WTcont2_: mean = 27.2 ± 2.1 g (range: 23.1–31.3 g); weight _WTno dive:_ mean = 27.2 ± 1.9 g (range: 24.9–32.6 g)] (KW_KO-like/WTflux,/KO-like flux/WTcont2/WTno dive_, *n* = 27/24/24/21/17, *p* = 0.121). These weights were significantly higher than those of the flux-pool (KW_Allgroups_, *n* = 27/24/24/21/17/45/46/30/46, *p* < 0.0001), which may increase susceptibility to DCS.

Hence, in order to avoid confusion, results of both pools, i.e., flux and spad, are presented separately.

### Clinical observations

Compared to WT mice, the KO or KO-like mice showed no abnormal behavioral or phenotypic signs. All groups displayed a similar range of DCS symptoms (Figure 1).

Most of the mice were prostrate, suggesting that they were in physiological distress. Symptoms of DCS or death occurred after returning to the surface. Mice died from convulsions (on the outer edge of the radar map, Figure 1) and/or respiratory distress. Mice essentially displayed neurological symptoms with varying degrees of severity with motor and locomotor impairments (paraplegia, paraparesis) and, in some cases, convulsions. We also observed fewer convulsions and slightly more labored breathing in KO-like_flux_ (Figure 1B Radar chart). The opposite trend was found for WT_cont2_ (Contingency table, $\chi_{{}_{\text{KO-like/WTflux2/KO-like flux/WTcont2/WTno dive}}}^{2}=63.175$ vs. 21.026, *p* < 0.0001).

In the flux-pool, DCS symptoms generally occurred at 5.8 ± 3.5 min on average after the end of the dive. When mice succumbed to DCS, it occurred rapidly, i.e., 6.1 ± 3.1 min on average. There was no significant difference for the onset of first symptoms or death latency, regardless of the group (KW_WTcont/WTflux/HETflux/KOflux_: *p* = 0.399, *p* = 0.390, respectively).

In the spad-pool, first DCS symptoms generally occurred at 6.2 ± 3.4 min on average after the end of the dive. When mice succumbed from DCS, it occurred more rapidly, at 2.1 ± 4.5 min on average. There was no significant difference in the onset of first symptoms or death, regardless of the treatment (KW _KO-like/WTflux2/KO-like flux/WTcont2/WTno dive_: *p* = 0.620, *p* = 0.550, respectively).

### Lethal DCS, Grip−, Grip+ status

KO_flux_ and KO-like_flux_ mice were less susceptible to the hyperbaric protocol than other groups. Fewer KO-like mice succeeded in the grip tests (Figure 1 histograms).

Analysis of the different clinical status of DCS showed a significant difference between the WTcont group and WTflux, HETflux, and KOflux groups (KW_WTcont/WTflux/HETflux/KOflux_: *n* = 45/46/30/46, α = 0.05, *p* < 0.0001) (Figure 1; Table 1). Only 7% of KO_flux_ mice died as a consequence of DCS. Additionally, KOflux mice presented a better success rate in both their grip tests (Contingency table, $\chi_{{}_{\text{WTcont/WTflux/HETflux/KOflux}}}^{2}=39.194$ vs. 16.919, *n* = 45/46/30/46, α = 0.05, *p* < 0.0001).

**Table 1:** **Clinical status after a dive**.

**Populations**		**Clinical status after the hyperbaric exposure**		
		**Grip+(%)**	**Grip−(%)**	**Lethal DCS(%)**
Flux-pool	WT cont	22	16	62
	WT flux	46	13	41
	HET flux	60	23	17
	KO flux	78	15	7
Spad-pool	WT cont2	52	19	29
	KO-like	26	33	41
	KO-like flux	54	42	4
	WT flux 2	46	38	17

*Survivors underwent two grip tests. Grip tests, i.e., motor/sensory tests, were used to quantify forelimb involvement 15 and 30 min after the end of decompression. Mice which failed at least one test were considered to be symptomatic (Grip−). The results of this behavioral test were used to define DCS and distinguish the following groups: dead (Lethal DCS), mice that failed at least one grip test (Grip−) and mice that passed both grip tests (Grip+)*.

In the spad-pool, analysis of the different DCS statuses showed a significant difference between the different groups (KW _KO-like/WTflux2/KO-like flux/WTcont2/WTno dive_: *p* < 0.0001). Interestingly, a significant difference appeared between the KO-like group and the KO-like_flux_ group. The main difference corresponded to the number of animals that died from DCS, i.e., 41 and 4% for the KO-like group and KO-like_flux_ group, respectively (Figure 1; Table 1). Surprisingly, the number of mice that died was lower in the KO-like_flux_ group than in the WT_flux2_ group, i.e., 4 and 17% respectively (Figure 1; Table 1). The success rate in both grip tests (Grip+) was lower for the KO-like group (Figure 1; Table 1; Contingency table, $\chi_{{}_{\text{KO-like/WTflux2/KO-like flux/WTcont2/WTno dive}}}^{2}=14.496$ vs. 15.507, *p* = 0.070).

### Mice that failed the grip test (Grip−)

Survivors underwent two grip tests (Figure 1; Table 1). While results of grip tests were different according to the treatment group, no treatment seems to better promote recovery or worsen the physical state of mice once symptoms become manifest. We previously suggested that the absence of TREK-1 channel activity could limit recovery after DCS (Vallee et al., 2012), but our present data are not in accord with such a hypothesis. For example, a large number of KO_flux_ mice passed both grip tests, but without an improvement between both tests, whereas KO-like_flux_ mice not only passed both grip tests, but improved their scores between test 1 and test 2.

In more detail and with regard to the timed performance in the grip tests, WT_flux_ mice improved their mean time spent suspended from the cord (W_WTflux_: *n* = 6, α = 0.05, *p* = 0.034) in the second test carried out 15 min later (16.8 ± 2.1s vs. maximal time 30.0 ± 0.0 s). In the spad-pool study, better performances were observed in the second grip tests for the KO-like population (W_KO-like_: *p* = 0.014, 4.1 ± 5.8 s vs. 16.0 ± 11.2 s) and the KO-like_flux_ population (W_KO-like flux_: *p* = 0.014, 11.5 ± 8.9 s vs. 22.2 ± 11.1 s).

Significant differences were observed between populations in both grip tests (first test, KW_WTcont/WTflux/HETflux/KOflux_:*n* = 7/6/7/7, α = 0.05, *p* = 0.048 and second test, KW _WTcont/WTflux/KOflux_: *n* = 7/6/7/7, α = 0.05, *p* = 0.039): the WT_cont_ group did not perform as well as mice treated with fluoxetine (first test: MW _WTcont/KOflux_: *n* = 7/7, *p* = 0.054, MW _WTcont/WTflux_: *n* = 7/6, *p* = 0.002; second test: MW _WTcont/WTflux_: *n* = 7/6, *p* < 0.0001). WT_flux_ mice performed better in the second grip test than HET_flux_ mice (MW_HETflux/WTflux_: *n* = 7/7, *p* < 0.009). With regard to timed performance in the grip tests of the spad-pool, no significant differences were observed between populations, either in the first (KW: *p* = 0.190) or second grip test (KW: *p* = 0.453), or in their delta performance (KW: *p* = 0.193).

### Full blood counts

#### Erythrocyte counts

To a small extent, a decrease in red cell counts, usually though diapedesis, can be observed after a dive and can be attributed to the decompression protocol (Table 2). Overall, we cannot link this decrease to an effect of treatment or to mouse genotype.

**Table 2:** **Variation (%) in erythrocyte, leukocyte, and platelet counts before and after decompression**.

**Correction according to the hematocrit variation**	**Part of the study**	**Variation in blood cell counts between before and after the hyperbaric exposure (%)**		
		**Grip/+**	**Grip/−**	**All survivors**
Hematocrit	1-Flux-pool	−14.4±19.2	−4.5±25.3	−12.6±21.6^*^
	2-Spad-pool	+ 29.9±51.8	+ 31.0±48.3	+ 25.2±43.8^*^
Erythrocytes	1-Flux-pool	−0.6±8.9	0.2±1.8^#^	−0.4±7.9^*^
	2-Spad-pool	−7.3±9.4	−10.2±11.0	−8.6±10.1^*^
Leukocytes	1-Flux-pool	−6.6±66.6	+ 12.1±80.8	−2.4±69.7^*^
	2-Spad-pool	−29.0±51.7	−25.4±74.7	−27.4±62.8^*^
Platelets	1-Flux-pool	+ 9.2±33.1	−13.6±29.2^#^	+ 4.5±33.5
	2-Spad-pool	−26.1±23.8	−41.2±29.3	−32.7±27.0^*^

*The column on the right shows the averaged count carried out for all survivors (Grip+ and Grip−). Grip− mice were considered to be symptomatic. Grip+ mice were considered to be asymptomatic*.

* *Denotes a significant difference within all the survivors between pre- and post-decompression counts*.

# *Denotes a significant difference between Grip+ and Grip− mice*.

In the flux-pool, differences in erythrocyte counts could be attributed to the treatment before the dive (KW: *p* < 0.0001): WT_cont_ mice displayed lower erythrocyte counts than both WT_flux_ (*p* < 0.001) and KO_flux_ (*p* < 0.003) mice. HET_flux_ mice also presented lower erythrocyte counts than KO_flux_ mice (*p* = 0.01). Globally, erythrocyte counts decreased on average after the dive, to a small extent (W: *p* = 0.003, mean = −0.4 ± 7.9), with lower counts in Grip− mice (KW *p* = 0.017). These differences can therefore be related to treatments given to the mice (KW *p* < 0.0001: WT_cont_ and WT_flux_ presented lower erythrocyte counts than KO_flux_), but there were no variations in their consumption rate (KW *p* = 0.382) after the dive. This suggests that the red cell count decrease was due to the decompression, even when basal levels were different according to treatment.

In the spad-pool, no effect on erythrocyte counts could be attributed to the sole effects of treatments before the dive (KW: *p* = 0.999), or afterwards (KW: *p* = 0.392), or in its variation between before and after the dive (KW: *p* = 0.172). Following the dive, a significant decrease in erythrocyte counts (W *p* < 0.001, mean = −8.6 ± 10.1) was observed after the decompression protocol in all survivors, but the variation was not linked to the clinical state after the dive (Grip+ vs. Grip−: MW *p* = 0.299: Grip+: mean = −7.3 ± 9.4%; Grip−: mean = −10.2 ± 11.0%).

### Platelet counts

Globally, platelet loss occurs following a dive that can be linked to the physical state of mice. Platelet consumption seemed to be more related to the clinical state of the mice than to their treatment or their genetic status (Table 2). Platelet consumption increased with the severity of DCS. This drop in platelet counts was attributed to clotting activity following exposure of the collagen under bubble-damaged endothelial cells in blood vessels (Persson et al., 1978; Haller et al., 1987; Thorsen et al., 1987; Nossum et al., 1999) or to direct interactions between bubbles and platelets (Hallenbeck et al., 1973; Warren et al., 1973; Giry et al., 1977).

In more detail, and with regards to the first part of the study, some differences in platelet counts could be attributed to the treatments before the dive (KW: *p* < 0.001): WT_cont_ displayed lower platelet counts than both HET_flux_ (*p* = 0.004) and KO_flux_ (*p* < 0.001). WT_flux_ also presented lower platelet counts than HET_flux_ (*p* = 0.004) and KO_flux_ (*p* < 0.001).

Following the dive, no significant decrease in platelet counts (mean = +4.5 ± 33.5%) was recorded when all survivors were considered (W *p* = 0.728). Nonetheless, Grip− mice tended to exhibit higher consumption of platelets than Grip+ mice (KW, *p* = 0.005; Grip+: mean = +9.2 ± 33.1%; Grip−: mean = −13.6 ± 29.2%). No link could be established between platelet consumption and treatments (KW, *p* = 0.076). This suggests that platelet consumption, attributed to clotting activity, is mainly due to the clinical state induced by decompression rather than to treatment or genotype.

In the spad-pool, the absence of a difference in platelet counts could be attributed to the treatment effects before the dive (KW: *p* = 0.209), and levels before the dive had no consequences on the clinical state after the dive (KW: *p* = 0.735).

Thirty minutes after the dive ended, a significant decrease in platelet counts (−21.2 ± 26.5%) was recorded in all survivors (MW: *p* < 0.0001). Platelet consumption was independent of the nature of the treatment (KW: *p* = 0.132). Grip+ mice had more circulating platelets (KW, *p* = 0.042) and tended to have a lower consumption (in proportion) of their platelets than Grip− mice (KW, *p* = 0.069; Grip+: mean = −26.1 ± 23.8%; Grip−: mean = −41.2 ± 29.3%). Actually, Grip− platelets had a greater volume than that of Grip+ mice (*p* = 0.0014), confirming that old platelets (with a lower normal volume) had been used in the aggregation process.

#### Leukocyte counts

Globally, there was a loss of leukocyte following the dive (Table 2) that was usually attributed to diapedesis (Dutka et al., 1989; Zamboni et al., 1989, 1992, 1993; Dal Palu and Zamboni, 1990; Helps and Gorman, 1991). Neither treatment seemed to influence leukocyte movement greatly, or the clinical state.

In the flux-pool, differences in leukocyte counts could be attributed to the treatments before the dive (KW: *p* = 0.001), as WT_cont_ displayed lower leukocyte counts than both HET_flux_ (*p* = 0.010) and KO_flux_ (*p* < 0.005). These differences influenced their clinical state after the dive (KW: *p* = 0.004). Therefore, mice that died after the dive-decompression protocol (Lethal DCS mice) had lower leukocyte counts before the dive than Grip+ mice (4.3 ± 1.2 vs. 5.3 ± 1.6 × 2.10^3^/μl).

After the dive, in all survivors, leukocyte counts decreased by 2.4 ± 7.9% (W: *p* = 0.003). These differences in counts or variation between the different groups were not significant with regard to genotype or treatment (KW: *p* = 0.129 and *p* = 0.342), or clinical status after the dive, i.e., Grip+, Grip−, or Lethal (KW *p* = 0.870 and *p* = 0.416).

In the spad-pool, differences in leukocyte counts could be attributed to the 5 days of spadin treatment (KW, *p* = 0.008). KO-like mice displayed higher leukocyte counts than KO-like_flux_ mice (*p* = 0.016), WT_flux2_ mice (*p* = 0.007) or WT_no dive_ mice (a trend, *p* = 0.096). Differences observed before the dive possibly influenced the clinical state after the dive (KW: *p* = 0.022). Here again, Lethal DCS mice had higher leukocyte counts than Grip+ mice (6.3 ± 1.7 vs. 5.0 ± 2.0 × 02.10^3^/μl, *p* = 0.032). Nonetheless, this is the opposite result to what has just been proposed for the flux-pool study, what must invalidate the hypothesis.

Following the dive, a significant decrease in leukocyte counts (W: *p* < 0.0001, mean = −27.4 ± 62.8%) was recorded in all survivors. Nonetheless, there was no difference in the consumption of leukocytes between Grip+ and Grip− mice (KW: *p* = 0.8930: Grip+: mean = −29.0 ± 51.7%; Grip−: mean = −25.4 ± 74.7%). Leukocyte recruitment was independent of the nature the treatment (KW: *p* = 0.905).

Finally, fluoxetine administered orally before the dive tended to increase the number of circulating leukocytes, compared to control mice. When injected i.p., fluoxetine reduced leukocyte counts. In fact, intraperitoneal injection of fluoxetine induces irritation and leukocyte adhesion in the abdominal cavity (Herr et al., 2014). This effect was not counteracted by the 5 days of spadin treatment, while the latter, when given alone i.p., tended to increase leukocyte counts. Finally, however, this prestimulation was equal in all groups for the spad-pool and controlled by the administration of the vehicle in the same way as fluoxetine administration. These paradoxical effects on leukocyte counts before the dive did not influence the (high) survival rate of the KO_flux_ and the KO-like_flux_ mice.

As already described (Pontier et al., 2008; Blatteau et al., 2012; Vallee et al., 2012), the leukocyte counts dropped after decompression in all survivors, and leukocyte recruitment was independent of the type of treatment.

### Blood biochemistry

Following the dive, blood biochemistry was assessed on samples from heart punctures. Compared to WT_no dive_, sodium (KW: *p* = 0.007; *post-hoc p* = 0.019 *p* = 0.01), potassium (KW: *p* = 0.022; *post-hoc p* = 0.006 *p* = 0.004), globulin (KW: *p* = 0.01; *post-hoc p* = 0.008 *p* = 0.001), and transaminase (KW: *p* = 0.001; *post-hoc p* = 0.002 *p* < 0.0001) were found at increased levels in mice subjected to the hyperbaric protocol, regardless of the clinical state (Grip+ or Grip−) (Table 3). Grip− mice had higher levels of creatinine kinase (KW *p* = 0.062; *post-hoc p* = 0.019). The BUN level was lower in the Grip+ groupthan in the WT_no-*dive*_ group (KW *p* = 0.041; *post-hoc p* = 0.016). The albumin level was lower in the Grip+ and the Grip− groups than in the WT_no-*dive*_ group (KW: *p* < 0.001; *post-hoc p* = 0.002 *p* < 0.0001). Triglyceride levels were higher in Grip− than in Grip+ mice (KW *p* = 0.005; *post-hoc p* = 0.01). Overall, the dive induced a hemoconcentration regarding sodium potassium and AST levels. This could be due to a capillary leak consider low albumin levels (in contrast to high globulin levels). Cell destructions and/or liver shouting could also be engaged, especially when taking into account high triglycerides levels then recorded in Grip−. Increase in creatinine kinase also suggested muscle or heart straining when DCS was stated.

**Table 3:** **Blood biochemistry analysis according to clinical status after the decompression protocol**.

**Effect of clinical status on:**	**KW**	***Post-hoc*** **test**	
Total bilirubin	0.004	Lethal DCS < WT_no dive_	*p* = 0.002
		Lethal DCS < Grip+	*p* < 0.0001
		Lethal DCS < Grip−	*p* = 0.002
Na+	0.007	WT_no dive_ < Lethal DCS	*p* = 0.001
		WT_no dive_ < Grip+	*p* = 0.019
		WT_no dive_ < Grip−	*p* = 0.01
		Grip+ < Lethal DCS	*p* = 0.031
		Grip− < Lethal DCS	*p* = 0.006
K+	0.022	WT_no dive_ < Grip+	*p* = 0.0006
		WT_no dive_ < Grip−	*p* = 0.004
		WT_no dive_ < Lethal DCS	*p* = 0.028
BUN/Urea	0.041	WT_no dive_ > Grip+	*p* = 0.016
AST/transaminase	0.001	WT_no dive_ < Grip+	*p* = 0.002
		WT_no dive_ < Grip−	*p* < 0.0001
Globulin	0.010	WT_no dive_ < no DCS	*p* = 0.008
		WT_no dive_ < DCS	*p* = 0.001
Lactate	0.016	Grip+ < Lethal DCS	*p* = 0.010
Creatinine kinase	0.062	WT_no dive_ < Grip+	*p* = 0.019
Triglycerides	0.005	Lethal DCS > Grip+	*p* = 0.005
		Grip+ > Grip−	*p* = 0.010
Albumin	0.000	WT_no dive_ > Grip+	*p* = 0.002
		WT_no dive_ > Grip−	*p* < 0.0001

Blood sample analyses from two mice, punctured just before they died (Lethal DCS) showed (i) higher levels of sodium (KW *p* = 0.007; *post-hoc p* = 0.001), potassium (KW *p* = 0.022; *post-hoc p* = 0.028) or creatinine kinase (KW *p* = 0.062; *post-hoc p* = 0.064) in comparison with WT_no dive_ mice, (ii) a higher sodium level in comparison with both the Grip− and Grip+ groups (KW *p* = 0.007; *post-hoc p* = 0.006 and *p* = 0.031 respectively), (iii) a higher lactate level (KW *p* = 0.016; *post-hoc p* = 0.010) and triglyceride concentration (KW *p* = 0.005; *post-hoc p* = 0.005) when compared to the Grip+ group, (iv) higher bilirubin levels than those of WT_no dive_, Grip+ and Grip− mice (KW *p* = 0.004; *post-hoc p* = 0.002 *p* < 0.001 *p* = 0.002, respectively), (v) unchanged glycemia, calcium, cholesterol, creatinine, and total protein levels vs. the other groups.

When looking at the effect of the treatments *per se* on blood biochemistry after the dive (Table 4), we pointed out that mice treated with fluoxetine, such as KO-like_flux_ and WT_flux2_ and corresponding to the best survival rates, had higher levels of potassium (hemoconcentration, cell destruction), AST (aspartate aminotransferase, liver function) and globulin (hemoconcentration), and lower levels of albumin (an indicator of capillary leak) and lactate (high levels indicate anaerobic) than those of the WT_no-*dive*_ group.

**Table 4:** **Blood biochemistry analysis depending on treatment after the decompression protocol**.

**Treatment effects on:**	**KW**	***Post-hoc*** **test**	
Albumin	0.001	WT_no dive_ > KO-like_flux_	*p* = 0.001
		WT_no dive_ > WT_flux2_	*p* < 0.0001
Na+	0.027	WT_no dive_ < KO-like	*p* = 0.0004
K+	0.000	WT_no dive_ < KO-like_flux_	*p* < 0.0001
		WT_no dive_ < WT_flux2_	*p* = 0.002
Aspartate T/transaminase	0.000	WT_no dive_ < KO-like_flux_	*p* < 0.0001
		WT_no dive_ < WT_flux2_	*p* < 0.0001
Total bilirubin	0.000	KO-like < KO-like_flux_	*p* < 0.0001
		KO-like < WT_flux2_	*p* < 0.0001
		KO-like < WT_no dive_	*p* = 0.002
Globulin	0.001	WT_no dive_ < KO-like_flux_	*p* = 0.001
		WT_no dive_ < WT_flux2_	*p* = 0.001
Lactate	0.014	KO-like > WT_flux2_	*p* = 0.003
Triglycerides	0.029	KO-like > KO-like_flux_	*p* = 0.004
		KO-like > WT_flux2_	*p* = 0.001

KO-like mice, receiving spadin for 5 days, displayed the worst survival rate. In comparison with the WT_no-*dive*_ group, they expressed very low levels of bilirubin, a toxic and hydrophobic breakdown product of red blood cells that is bound to and carried by albumin. They also had very high levels of lactate (anaerobic metabolism and possible mitochondria dysfunction), sodium and triglycerides; the origin of these high triacylglycerol levels in their blood could be due to adipocyte release, pancreatitis, autoimmune disease or nephritic syndrome.

## Discussion

In this study, fluoxetine should be regarded as an anti-inflammatory drug, considering that we opted for an acute, high dose of 50 mg/kg; the antidepressant effect of fluoxetine requires long-term treatment (28 days at 20 mg/kg) to induce synaptogenesis, and should not be considered in this study. In contrast, 5 days of spadin treatment was sufficient to internalize TREK-1 channels and consequently to exert antidepressant activity (Mazella et al., 2010; Moha Ou Maati et al., 2012).

### Prevention of decompression sickness

The decompression protocol used in this study is comparable with that used in previous studies on mice of a similar weight (Berghage et al., 1979; Blatteau et al., 2012; Vallee et al., 2012). Mice in the spad-pool were heavier, which may have increased their susceptibility to DCS. Nonetheless, the protocols used in this study induced neurological DCS with motor and locomotor impairments and convulsions, suggesting damage to the spinal cord or brain.

The increase in AST (aspartate aminotransferase) may be due to fluoxetine hepatotoxicity, as fluoxetine is extensively metabolized in the liver to norfluoxetine and this is a well-known side effect (Inkielewicz-Stepniak et al., 2014). Fluoxetine may also induce moderate lymphocyte infiltration within the portal tracts and ballooning degeneration of hepatocytes (Kwak et al., 2000). The low mortality associated with fluoxetine treatment led us to conclude that increased AST levels may have a minor effect on DCS outcomes. Nonetheless and except for Lethal DCS mice, we also observed increased concentrations of sodium and potassium ions and globulins, after the dive. These variations may be explained at least in part by hemoconcentration (increased hematocrit in the spad-pool) or by hepatocyte damage due to the presence of bubbles in the liver, as previously observed in rats (L'Abbate et al., 2010). Arguing in favor of liver dysfunction, the albumin and BUN (both synthetized by the liver) concentrations were low, but these lower concentrations could also been explained by damage to the glomerulus, as occurs in nephritic syndrome (Blann and Ahmed, 2014), or by capillary leak that may lead to edema (Clarkson et al., 1960). This albumin loss demonstrated that fluoxetine did not prevent capillary leak *per se*, a symptom of severe DCS. Finally, the blood biochemistry results indicate that damage induced by bubbles may have induced capillary leak, cell destructions and liver dysfunction. However, other blood components were not affected by the dive. Consequently, additional data will be necessary to validate or invalidate the liver dysfunction hypothesis. Dramatically, multivisceral dysfunction could be supported by changes in analyte concentrations in Lethal DCS mice, such as high levels of creatinine kinase or lactate.

This protocol also decreased erythrocyte, leukocyte and platelet counts, suggesting diapedesis, inflammation and clotting activity, as well as ischemia, the usual symptoms described for DCS. Platelet dysfunction has been described for fluoxetine (Lesch et al., 1993; Brenner et al., 2007), but this effect did not influence platelet recruitment or DCS outcomes in our study. On the other hand, TREK-1 channels are not known to affect clotting function directly, although they are activated by platelet activating factor (Maingret et al., 1999). Under these conditions, TREK-1 channels likely played a minor role in DCS outcomes.

The main result from our work is that mice with impaired TREK-1 channel function and treated with fluoxetine are more resistant to the consequences of decompression than WT mice treated with fluoxetine, and far more resistant than untreated KO or KO-like mice. Our data suggest that the presence of the TREK-1 channel may mitigate the global benefit of fluoxetine in DCS-related ischemia and inflammation. The second important result from our work is that 5 days of spadin antidepressant treatment, thereby blocking TREK-1 channel activity specifically, increased DCS susceptibility. However, when the same 5 days of spadin treatment was administered to mice treated with fluoxetine, the protective effect of fluoxetine was increased. These data indicate that the main protective effect of fluoxetine is mediated by a cellular pathway independent of TREK-1 channel activity.

### Loss of protective activity of TREK-1

The high mortality rate of KO-like mice confirmed that the neuroprotection induced by the TREK-1 channel in WT was prevented by spadin (5 days of treatment). The importance of the TREK-1 channel in neuroprotection is well-documented for different physio-pathological processes, including hypoxia (Moha Ou Maati et al., 2011), glutamatergic excitotoxicity and neuronal death (Heurteaux et al., 2004). According to our previous and present studies (Vallee et al., 2012), mice that do not express the TREK-1 channel (KO-like or KO) are more likely to develop neurological symptoms after DCS damage to the central nervous system. This could be attributed to the stimulation of NMDA receptor activity, which exacerbated glutamate excitotoxicity, making the brain blood barrier (BBB) more permeable to leukocytes (Bittner et al., 2014). These KO mice are known to display an inflammatory phenotype and accelerated leukocyte trafficking across the BBB (Bittner et al., 2014).

This study focused on fluoxetine, which inhibited TREK-1 channel activity (Kennard et al., 2005; Sandoz et al., 2011) and thus reduced its neuroprotective effects. However, in the present work and in previous experiments on fluoxetine and DCS (Blatteau et al., 2012), we found that WT mice treated with fluoxetine were more resistant to DCS than untreated mice. These observations suggest that the positive anti-inflammatory effects of fluoxetine outweigh fluoxetine-induced TREK-1-inhibition.

### Enhanced protective activity of fluoxetine on TREK-1^−/−(like)^ mice

**Figure 2:**
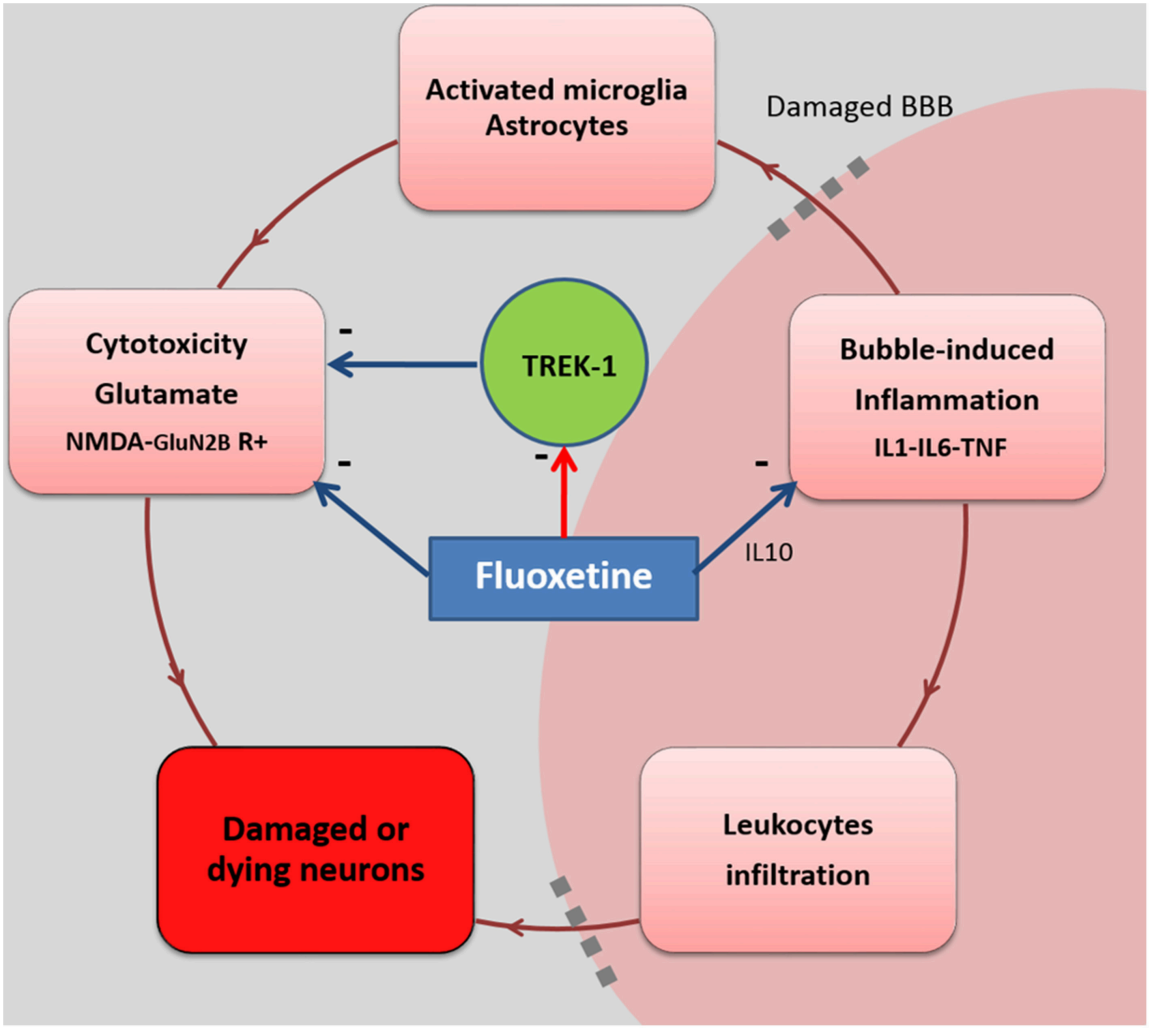
**Fluoxetine and possible interactions in a CNS-ischemia model**. The circle summarizes the deleterious cascade encountered after a provocative dive, resulting from vascular bubble formation. Fluoxetine stimulates IL-10 secretion which attenuates the inflammatory response mediated by IL-1, IL-6, and TNF. This reduces leukocyte infiltration across the BBB. Fluoxetine is also known to selectively block glutamate N2B-containing NMDA receptors (non-synaptic 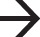 neurodegenerative), reducing the negative effects of excitotoxicity on neurons. However, blockade of the TREK-1 channel by an SSRI such as fluoxetine may contribute to the limitation of the protection afforded by these channels, but it may also strengthen the fluoxetine action at other sites in a dose-dependant manner.

KO and KO-like mice treated with fluoxetine appeared to be the most resistant to decompression. It was recently shown that fluoxetine selectively inhibits glutamate-N2B NMDA receptors and then induces neuroprotection (Vizi et al., 2013). This property of fluoxetine could partly substitute for the loss of TREK-1-mediated neuroprotection. Another hypothesis can also be drawn. In the absence of TREK-1 channels at the cell membrane, more fluoxetine may have been available for binding to other fluoxetine targets, thereby increasing its protective effects. This hypothesis is suggested by the dose dependence of DCS in KO_flux_ HET_flux_ and WT_flux_ mice treated with fluoxetine. We previously attributed the main protective effect of fluoxetine in DCS to its anti-inflammatory properties from the observed decrease in IL-6 (Blatteau et al., 2012). Decreased IL-6 secretion was also described in TREK-1-deficient cells (Schwingshackl et al., 2012) and this could superimpose on the effect of fluoxetine to further reduce inflammation in TREK-1 deficient mice. It could constitute an efficient response to TREK-1^−/−^-induced BBB damage (Bittner et al., 2013, 2014) or more hypothetically, an opportunity to cross the BBB more readily and more efficiently (Figure 2). Finally, fluoxetine is also known to prevent BBB disruption (Lee et al., 2014) thanks to its anti-inflammatory activity coupled with its ability to block NMDA receptors and attenuate glutamatergic excitotoxicity. Further studies will be required to confirm this hypothesis.

### Should SSRI treatment be a contraindication for diving?

Spadin was described as a new concept of antidepressant and it was shown that 4 days of treatment with spadin corresponds to 21–28 days of treatment with classical SSRIs (Mazella et al., 2010; Moha Ou Maati et al., 2012; Borsotto et al., 2015; Devader et al., 2015; Veyssiere et al., 2015). Spadin treatment induces TREK-1 internalization, resulting in the disappearance of the channel from the plasma membrane (Mazella et al., 2010). KO mice are known for developing a depression-resistant phenotype and also for being more sensitive to ischemia (Heurteaux et al., 2006). Both KO and KO-like TREK-1 mice were more likely to succumb to DCS after dive. Therefore, we legitimately wonder whether SSRIs, in their usual indication against depression in humans, could represent a risk factor in diving, or more generally in risky sports that may induce ischemia and inflammation.

### Fluoxetine, TREK-1 and clinical status: weight

TREK-1 is widely expressed in central and peripheral tissues. It is highly expressed in the nervous system, digestive system, endocrine system and reproductive system, as well as in the muscular system. In the CNS, TREK-1 is expressed in both astrocytes and neurons. It should be kept in mind that fluoxetine, like spadin, could spread throughout whole body and thus could induce many interactions not anticipated in this study, even if the symptoms are mainly nervous. Moreover, it could be useful to include in further studies immuno-histological technics to confirm the infiltrating leukocyte population in the brain injury. The signaling pathways should also be of interest to assess cell viability.

## Conclusion

We previously found that acute fluoxetine treatment affords the best protection against DCS when the TREK-1 potassium channel is impaired. We suggest that this drop in mortality may be due to decreased ischemia-induced glutamatergic toxicity, which could be due to the blockage of NMDA receptors by fluoxetine, and/or to an enhancement of the anti-inflammatory effect of fluoxetine. Following this study, exact mechanism remains to be elucidated. We also found that antidepressed mice, i.e., KO or spadin treated, showed increased susceptibility to DCS compared to WT mice. It remains to be determined whether antidepressants could be contraindicated in humans participating in risky activities such as diving that may induce ischemia, even though we found an efficient but paradoxical solution to DCS by delivering fluoxetine.

## Author contributions

JB and NV: conception and design of research; JB, SM, KL, PR and NV: performed experiments; JB, KL, and NV: analyzed data; JB, KL, SM, JM, MB, CH, JA, JR, and NV: interpreted results of experiments; NV: prepared figures; JB: drafted manuscript; NV: edited and revised manuscript; JB, KL, SM, JM, MB, CH, JA, JR, and NV: approved final version of manuscript.

## Funding

The work should be attributed to the “Institut de Recherches Biomédicales des Armées” laboratories. It is supported by Grant No. PDH-1-SMO-2-722 from the Délégation Générale pour l'Armement of the French Army, Paris, France, and by a special Grant from “La fondation des gueules Cassées,” Paris, France.

### Conflict of interest statement

The authors declare that the research was conducted in the absence of any commercial or financial relationships that could be construed as a potential conflict of interest. The Reviewer AG declares that, despite sharing a common affiliation with the Guest Associate Editor, the review process was carried out objectively and no conflict of interest exists.
